# 

**Published:** 1913-02-15

**Authors:** 


### ANNOUNCEMENTS.

CHICAGO DENTAL SOCIETY. At the annual meeting of the Chicago Dental Society held at 31 W. Lake St., Chicago, the election of officers resulted as follows: President, Geo. N. West; Vice-President, P. G. Puterbaugh; Secretary, T. L. Grisamore; Treasurer, F. E. Roach; Librarian, E. D. Coolidge.

THE PANAMA PACIFIC DENTAL CONGRESS. As one of the attractions of the Panama Pacific International Exposition, a Dental Congress, international in character, to be known as the Panama Pacific Dental Congress, is to be held in San Francisco, California, beginning on the last Monday in August, 1915, and continuing for ten days.

A Committee of Organization has been perfected, including representatives from the Pacific Coast States—California, Oregon, Washington, Utah, Idaho, Colorado and Arizona.

This committee is now actively engaged in perfecting the work of organization, including the establishment in every State of the United States and every foreign country, where dental organizations are known to exist, of Executive Committees, which will be empowered to promote the business of the Congress by bringing it to the attention of their National, State and Local Societies, and securing memberships and contributions to the program.

The American Society of Orthodontists and the National Dental Association, of the United States of America, have already made arrangements to meet in San Francisco in 1915 as parts of the Congress, and invitations will be extended to other dental societies to take similar action.

The Panama Pacific Dental Congress is the first organization to apply to the Exposition management for space for exhibits and to ask that a definite time be set aside for its meeting.

Manufacturers of dental goods have signified their intention to maintain during the Congress the greatest exhibition of dental supplies ever held; ample space for this purpose has already been promised by the Exposition authorities, and we are assured of their hearty co-operation in all things pertaining to the success of the Congress.

The membership fee has been fixed at ten dollars, and the finances of the Congress are being cared for by a corporation formed within the Committee of Organization, and known as the "Pacific Dental Congress Commission of 1915."

Over $8000.00 has already been subscribed for promotion purposes by the dentists and dental societies of the Pacific Coast States, and this fund will be increased by many thousands of dollars before the Congress meets.

Ample funds for the promotion of the Congress are assured, and in due time Committees on Local Arrangements, Transportation, Exhibits, Clinics, Program, etc., will be appointed, and everything possible will be done to insure the success of the Congress and make it in attendance and scientific and professional interest the greatest dental congress ever held.

The whole world is coming to San Francisco in 1915 to participate in and enjoy the Panama Pacific International Exposition, which will commemorate the completion of the world's engineering masterpiece, the Panama Canal.

Never in the history of the profession has there been so auspicious a time for holding a great dental congress, and the Panama Pacific International Exposition Company and the Committee of Organization of the Panama-Pacific Dental Congress unite in a cordial invitation to the members of the dental profession to come to San Francisco in 1915 to attend the Congress and view the wonders of the Exposition and Pacific Coast of the United States of America.

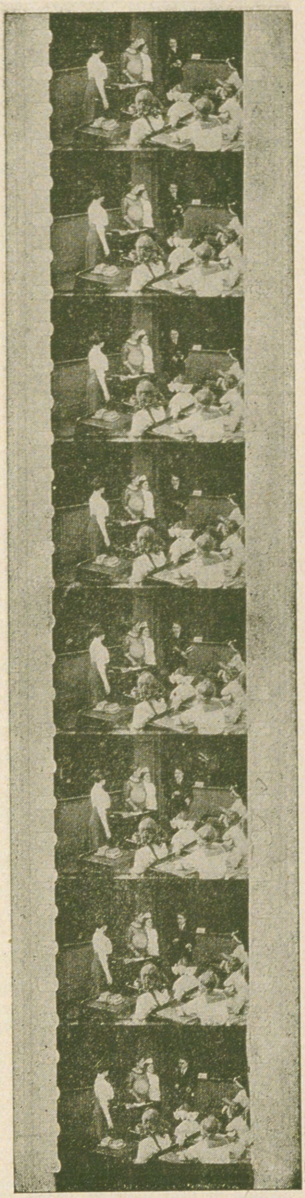

Section of film showing lecture to Class.

### DENTAL MOTION PICTURES.

PRODUCED UNDER THE AUSPICES OF THE NATIONAL MOUTH HYGIENE ASSOCIATION.

The "Toothache" Motion Picture Play tells a story of human interest combined with educational features that it would be impossible to convey to the public in any other conceivable way.

It shows the public how dental examinations are made in the public schools, the proper way to use floss silk, the tooth brush and how to masticate food.

It is designed to be added to the programme of the conventional motion picture theater, and in that way will be seen by countless millions of people throughout the world whom it would be impossible to educate in any other way.

The film is controlled by the film committee of the National Mouth Hygiene Association, hence may not be used for improper purposes, and aside from being sold to state boards of health, etc., will be sold only to regularly organized dental societies, or groups of ethical dentists, and may be rented or donated to motion picture houses or used in connection with lectures on the subject of oral hygiene in which every progressive dentist is or ought to be interested.

Complete scenario of the play will be furished on request, accompanied by a concrete and tangible plan whereby the cost of showing it may be reduced to the minimum or actually show a profit to the organization exploiting it.

Write for details.

George Edwin Hunt,
131 East Ohio Street,
Indianapolis, Ind.,

Sales Agent for the Committee.

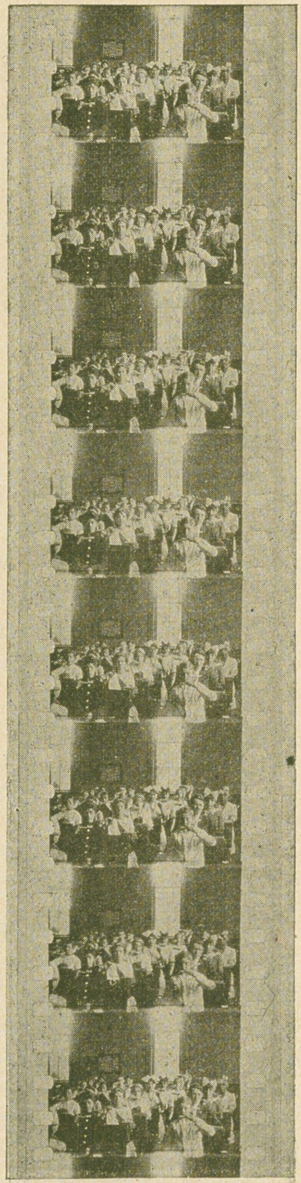

Section of film showing Tooth Brush Drill.

